# Assessment of microbial activity by CO_2_ production during heating oil storage

**DOI:** 10.1002/elsc.202100144

**Published:** 2022-04-21

**Authors:** Maximilian J. Surger, Lars M. Blank

**Affiliations:** ^1^ Institute of Applied Microbiology (iAMB) Aachen Biology and Biotechnology (ABBt) RWTH Aachen University Aachen Germany

**Keywords:** defined mixed culture, heating oil storage, microbial activity, microbial contamination, off‐gas‐analysis

## Abstract

Microbial activity is the driving force of the carbon cycle, including the digestion of biomass in the soil, oceans, and oil deposits. This natural diversity of microbial carbon sources poses challenges for humans. Contamination monitoring can be difficult in oil tanks and similar settings. To assess microbial activity in such industrial settings, off‐gas analysis can be employed by considering growth and non‐growth‐associated metabolic activity. In this work, we describe the monitoring of CO_2_ as a method for measuring microbial activity. We revealed that the CO_2_ signal corresponds to classical growth curves, exemplified by *Pseudomonas fluorescens*, *Yarrowia lipolytica*, and *Penicillium chrysogenum*. Deviations of the CO_2_ signal from the growth curves occurred when the yield of biomass on the substrate changed (i.e., the non‐growth‐associated metabolic activities). We monitored CO_2_ to track the onset of microbial contamination in an oil tank. This experimental setup was applied to determine the susceptibility of heating oil and biodiesel to microbial contamination long before the formation of problematic biofilms. In summary, the measurement of CO_2_ production by bacteria, yeasts, and molds allowed the permanent monitoring of microbial activity under oil storage conditions without invasive sampling.

**Abbreviations**
CDW: cell dry weight
HEL: extra light heating oil

## 1 INTRODUCTION

Microbial viability is the decisive measure for assessing the microbial contamination risk of products in the food or oil industry. The viability of microbial cells is usually determined by three parameters: membrane integrity, metabolic activity, and growth [1, 2, 3]. Membrane integrity may be an indirect measure of microbial viability. However, depending on the method of sterilization (e.g., UV irradiation, certain biocides, and pasteurization), dead cells possess intact membranes [1, 2]. An assessment of the risk of contamination based solely on the proliferation capacity provided by classical growth measurements is insufficient under certain environmental conditions, such as the maintenance of a cold chain. Microbes that do not currently proliferate may be metabolically active, have intact membranes, and have the ability to reproduce. By a small change in culture conditions, environmental signals, cell density, or microbial composition, viable, previously not or very slow‐growing microbes can promote contamination [3]. In particular, in the presence of complex microbial communities, the measurement of a single parameter does not provide a valid statement on the risk of microbial contamination [1, 4]. The alternative molecular markers for viability include ATP and RNA, which serve as a measure of biomass and metabolic activity, and ensure membrane integrity during purification. The available analysis kits allow simple culture‐independent testing across species applicability, and the lowest detection limits (ATP: 1 pmol/mL; RNA: 0.16–1.60 ng/μL) [5– 9].

**FIGURE 1:**
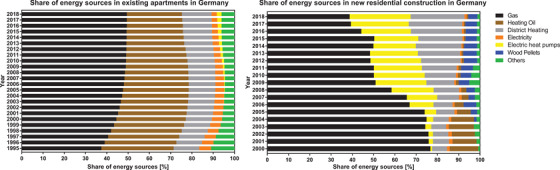
The spreading of heating oil burner systems in Germany. Left, the share of energy resources in existing apartments; Right, the share of energy resources in new constructions over the last years

Microbial contamination of heating oils is commonly observed, with a potential impact on the tank, filters, and pumps. Although the use of heating oil is declining, in Germany (Figure 1) ( https://www.bdew.de/energie/waermemarkt/ [accessed October 15, 2020]), there has been a push for the development of climate‐friendly alternative fuels that can be used in existing infrastructure. The problem of microbial storage stability persists and has even been exacerbated by the development of fatty acid methyl esters (FAMEs), one of the first fuel alternatives [10]. However, classical growth measurements and molecular alternatives cannot be applied in these two‐phase systems. The invasive sampling of individual locations in an oil tank covers only a defined time window and does not provide a complete picture of the contamination event. Furthermore, samples are compromised by the adherence of oil, which affects the feasibility of optical density and cell dry weight measurements, cell counting, and ATP and RNA assays.

**PRACTICAL APPLICATION**

The online monitoring of microbially produced CO_2_ and the associated experimental setup enable the continuous monitoring of microbial activity in two‐phase systems, such as the storage of petroleum products. Online monitoring requires no invasive sampling, no complex sample processing, and no direct contact between the sample matrix and measurement technology. All alternative classical and molecular methods for measuring microbial viability or contamination status are sensitive to organic sample matrices. The proposed approach is currently used to assess the microbial storage stability of fossil fuels and alternative fuels. This study is expected to support the development of microbial‐resistant blending strategies and antimicrobial additives.

CO_2_ formation can be monitored to serve as a method for determining microbial activity in heating oil tanks. Microbial CO_2_ production, the final product of microbial catabolic activity, was employed by Zhang et al. (1998) to assess diesel blend degradability [11, 12]. Recently, Rose et al. used the quantification of CO_2_, reduced to methane, by gas chromatography equipped with a flame ionization detector (GC‐FID), as a measure for the degradability of defined plastic monomers by individual bacteria in a mineral medium [13]. Individual gas samples could be directly measured without further processing. However, in both cases, the disadvantage of the defined time window remains.

Here, we opted to use continuous CO_2_ monitoring with IR sensors as an alternative. Through a comparison with classical growth curves, we demonstrated that CO_2_ online monitoring reproduces the growth of bacteria, yeasts, and molds. Nevertheless, the CO_2_ signal is based on the metabolic activity of the existing biomass. Therefore, deviations can occur due to changes in non‐growth‐associated metabolic activities. With the established setup, we used CO_2_ monitoring to assess the microbial susceptibility of heating oil and biodiesel immediately after production. The measurement of CO_2_ formation in real‐time can be easily applied to microbial contaminations that are difficult to access, including heating oil and plastic degradation.

## 2 MATERIALS AND METHODS

### 2.1 Microbial strains and growth conditions

Cultures of *Pseudomonas fluorescens* (*P. fluorescens* [14]) were grown in lysogeny broth (LB, 10 g/L peptone, 10 g/L yeast extract, 5 g/L NaCl). Single cultures of *Y. lipolytica* [14] were grown in yeast extract peptone (YEP, 20 g/L peptone, 20 g/L glucose, 10 g/L yeast extract) medium. Single cultures of *Penicillium chrysogenum* (*P. chrysogenum*, DSM 21171) were grown in malt extract (ME, 20 g/L malt extract, 1 g/L peptone) medium. Single strains in 100 or 200 mL of medium were cultured in 1 L Erlenmeyer flasks without baffles and were inoculated to an optical density (OD_600_) of 0.1 or 320 mg/L cell dry weight (CDW), respectively. Single cultures were shaken at 180 rpm.

**FIGURE 2:**
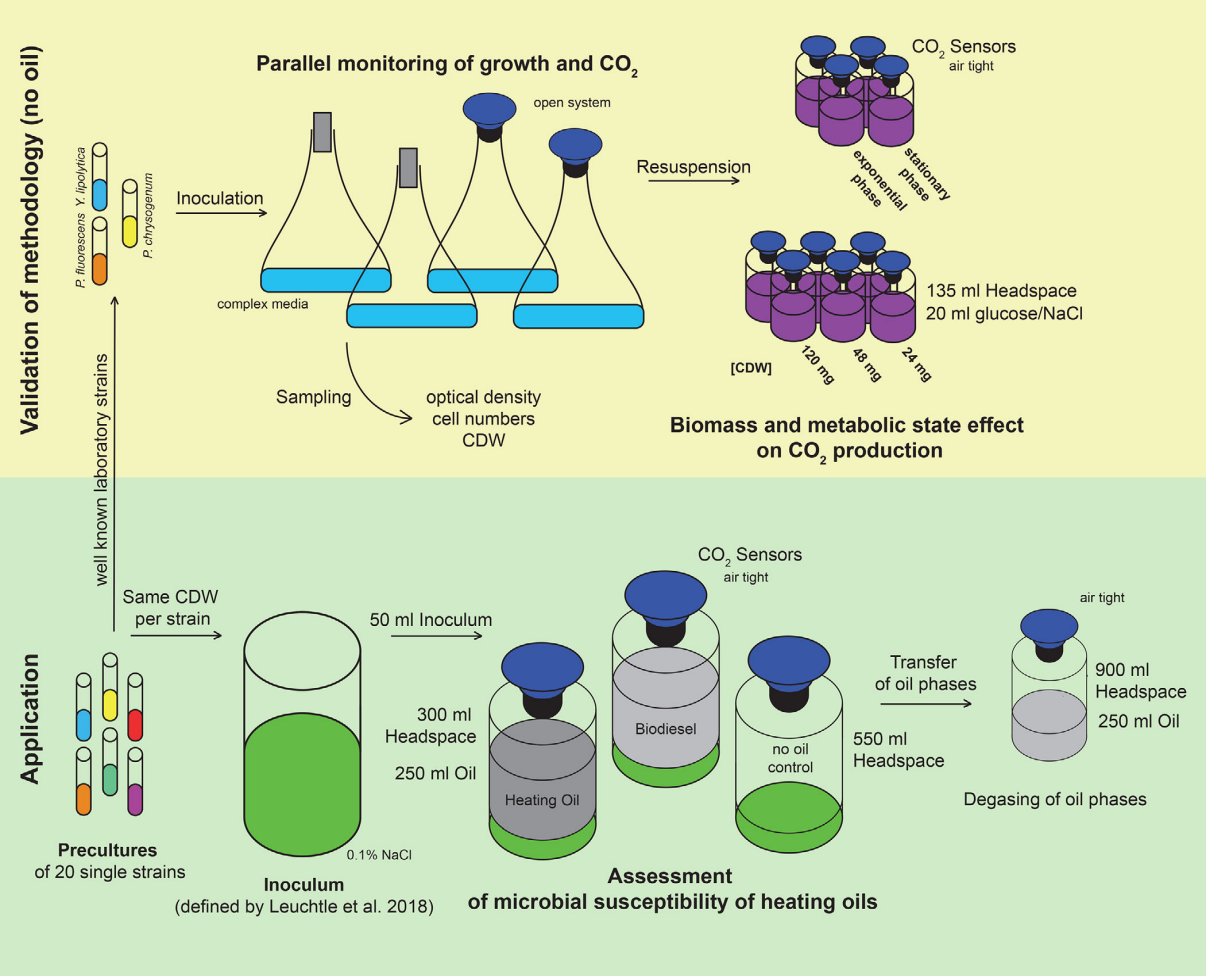
Overview of the experimental sections and setups of this study. The sections “Parallel monitoring of growth and CO_2_” and “Biomass and metabolic state effect on CO_2_ production” revealed the validation of the online monitoring of CO_2_ as a method for viability assessment. For these sections, three well known lab strains were used, which are also part of the later microbial mixture. In these sections, no oil phases were used, which enabled the application of classical growth measurements. In section “Parallel monitoring of growth and CO_2_,” complex medium was inoculated with one of three strains. On one‐half of the aerated shaking flasks, CO_2_ sensors were applied; from the other half of the shaking flasks, samples were collected for classical growth measurements. In section “Biomass and metabolic state effect on CO_2_ production,” cell samples of different growth phases were retrieved from the shaking flasks or different cell amounts were collected from the stationary phase, resuspended in glucose/NaCl solution, and sealed with a CO_2_ sensor in shot bottles. The application part shows the setup of the “Assessment of microbial susceptibility of heating oils.” The free water phase consisting of 0.1% NaCl was inoculated with equal amounts of 20 microbes, representative of heating oil (defined by Leuchtle et al. [14]). An aliquot was transferred into each storage culture and overlaid with heating oil, biodiesel or not (no oil control). The culture bottle was sealed airtight with a CO_2_ sensor. After completion of the storage cultures, the oil phases were transferred to new shot bottles and completely degassed to include the CO_2_ stored in the oil phase

To mimic an oil‐storage‐tank, 50 mL of free water phase with microbes was overlaid with approximately 250 mL heating oil or biodiesel within a 500 mL shot bottle, resulting in a 300 mL headspace. “No oil controls” consisted of a 50‐ml water phase with microbes and no oil phase, which led to a headspace of 550 mL (Figure 2). A volume of 800 mL water phase (0.1% NaCl) was inoculated with a mixture of 20 representative heating oil microbes (among others, *P. fluorescens*, *Y. lipolytica*, and *P. chrysogenum*) as defined by Leuchtle et al. [14], including an amount per strain corresponding to 16 mg CDW (a total of 320 mg for all microbes). The precultures were made in LB medium, YEP medium, potato extract glucose bouillon (PEGB, 26.5 g/L), and ME medium for bacteria, yeasts, *Rhodotorula mucilaginosa*, and molds. The precultures were washed with 0.1% NaCl before use. The microbial mixture did not contain anaerobes as the oil storage tanks were ventilated. Table 1 provides a summary of all strains. The bottles were not shaken and kept in the dark. All cultures (also oil storage simulations) used in this study were incubated at 25°C.

**TABLE 1:** Strains used in this study and the defined inoculum of the heating oil tank simulation [14]

**Strain**	**Database number**	**Source**
*Acinetobacter beijernickii*	DSM 22901	DSMZ
*Acinetobacter venetianus*	DSM 23050	DSMZ
*Burkholderia cepacia*	DSM 7288	DSMZ
*Burkholderia xenovorans*	‐	Leuchtle et al. [14]
*Micrococcus luteus*	‐	Leuchtle et al. [14]
*Micrococcus yunnanensis*	DSM 21948	DSMZ
*Pseudomonas fluorescens*	‐	Leuchtle et al. [14]
*Pseudomonas poae*	‐	Leuchtle et al. [14]
*Candida cylindracea*	DSM 2031	DSMZ
*Debaryomyces hansenii*	DSM 70244	DSMZ
*Debaryomyces polymorphus*	DSM 70816	DSMZ
*Pichia membranifaciens*	DSM 21959	DSMZ
*Raffaelea sp* .	‐	Leuchtle et al. [14]
*Rhodotorula mucilaginosa*	DSM 18184	DSMZ
*Ustilago maydis*	‐	Leuchtle et al. [14]
*Yarrowia deformans*	CBS 2071	CBS‐KNAW
*Yarrowia lipolytica*	‐	Leuchtle et al. [14]
*Paecilomyces lilacinus*	DSM 846	DSMZ
*Penicillium chrysogenum*	DSM 21171	DSMZ
*Penicillium citrinum*	‐	Leuchtle et al. [14]

### 2.2 Composition of the heating oil and biodiesel oil phases

#### 2.2.1 Extra light heating oil (HEL) of PCK refinery (Schwedt, Germany)

The extra light heating oil used in this study consisted of 62% aliphatic hydrocarbons and 38% aromatics with carbon numbers of C8‐C30. Detailed compositions are provided in Table 2.

**TABLE 2:** Composition of extra light heating oil (HEL) of PCK refinery (Schwedt, Germany) based on GCxGC‐MS by Laboratory Lommatzsch & Säger (Cologne)

**C‐Number**	**n‐/iso‐Alkanes**	**Cyclo‐alkanes**	**Mono‐Aromatics**	**Di‐Aromatics**	**Tri‐Aromatics**	**Tetra‐Aromatics**	**Total**
**C8‐C10**	1.5	1.2	0.3	0.0	0.0	0.00	**3.0**
**C11‐C15**	12.4	12.3	12.2	0.5	0.0	0.00	**37.4**
**C16‐C20**	11.6	10.3	9.6	5.2	0.0	0.01	**36.7**
**C21‐C25**	6.7	5.6	3.8	3.7	1.0	0.06	**21.0**
**C26‐C30**	0.5	0.6	0.3	0.2	0.2	0.11	**1.9**
**Total**	**32.7**	**30.1**	**26.2**	**9.5**	**1.3**	**0.18**	**100.0**

#### 2.2.2 Biodiesel

The biodiesel used in this study was produced from rapeseed oil (rapeseed oil methyl esters, RME), which mainly comprised methyl esters of oleic acid (C18:1) and to a lesser extent, methyl esters of linoleic acid (C18:2) and linolenic acid (C18:3).

### 2.3 Growth measurements

Measurements of optical density in oil‐free cultures were performed at a wavelength of 600 nm using a spectrophotometer (Ultrospec 10, Biochrom). Cell counting was performed using disposable C‐Chip Neubauer Improved chambers (NanoEntek) and a light microscope (ICC 50, Leica Microsystems). CDW was calculated by subtracting the weight of empty filters, which were incubated overnight at 120°C, from the weight of filters used for culture filtration that were also incubated overnight at 120°C. Glass fiber filters with 0.4 μm diameter were used (GF‐5, Macherey Nagel). Weight was measured using a moisture analyzer (MAC 50/1/NH, RADWAG).

### 2.4 CO_2_ measurement

To measure CO_2_ development, we used a BCP‐CO_2_ system (BlueSens Gas Sensor GmbH, Herten, Germany). The CO_2_ sensor inhabits a source of infrared light, which is weakened by the analyte gas and reflected into the detector unit of the sensor. The sensor was attached airtight to the opening of a culture vessel; this vessel was either a 100 mL shot bottle, a 500 mL shot bottle, or a 1 L Erlenmeyer flask without baffles. Measurements were performed in aerated 1 L Erlenmeyer flasks (further openings in addition to the airtight sensor attachment) for cultures of single bacteria, yeasts, or molds using complex media. Other measurements in 100 or 500 mL shot bottles were performed without air exchange. For the airtight 500 mL shot bottles used for the storage cultures in the section “Application of CO_2_ measurement for the assessment of the microbial susceptibility of heating oils” sealable valves were applied that allowed one CO_2_ sensor to be switched between two shot bottles. Alternating measurements of two replicates were possible.

### 2.5 Isolation of biomass and metabolic state effect on CO_2_ production

To demonstrate the biomass effect, cell amounts corresponding to 24 mg (10 mL of OD_600_ of 2.1), 48 mg (20 mL of OD_600_ of 2.1), and 120 mg CDW (50 mL of OD_600_ of 2.1) were collected from one stationary *P. fluorescens* culture (using a OD_600_ to CDW conversion factor of 1.15 $\frac{mg}{\left(OD_{600}\;\times \;ml\right)}$). To demonstrate the effect of the overall metabolic setting, equal amounts of cells (CDW of 46 mg each), normalized by optical density, were retrieved from one *P. fluorescens* culture in the exponential (38 mL of OD_600_ of 1.05) and stationary phases (15 mL of OD_600_ of 2.75), assuming two different defined metabolic states. The cells were washed with a glucose/NaCl solution (20 g/L and 0.9%) for 10 min at 5000 rpm and 4°C, and resuspended in 20 mL each. In the glucose solution, no growth was possible due to the absence of nitrogen and phosphorus sources. However, metabolic activity and CO_2_ production were based on the current enzymatic equipment. Because the same volume was used for resuspension, the same headspace volumes occurred below the CO_2_ sensors. The CO_2_ measurement was performed in 100 mL airtight shot bottles to measure the accumulation of CO_2_. The accumulation rate was calculated as the slope of the linear regression describing the CO_2_ accumulation during the first hours.

### 2.6 Degassing of the oil phases

After completion of the storage culture series, the oil phases were transferred to new 1 L shot bottles, and sealed airtight with the BCP‐CO_2_ sensors. Over the course of 24 h, a new CO_2_ equilibrium was established between the oil phase (250 mL) and headspace (900 mL). The headspace was rinsed with compressed air until the measured value reached 0.04% (v/v) (ambient air). Over the course of another 24 h, equilibrium was re‐established. The procedure was continued until the measured value in the headspace no longer reached 0.1% (v/v) CO_2_. The final values of the degassing cycles (minus 0.04% (v/v)) were summed up, converted into mg CO_2_, and compared with the final CO_2_ value of the storage culture series. As a result, a linear correction factor was calculated and applied for the entire duration of CO_2_ monitoring. A stable relationship/equilibrium between CO_2_ storage in the oil phase and CO_2_ release into the headspace was assumed over the entire culture duration.

### 2.7 Gas‐chromatography to assess oxygen depletion

The multiple gas analyzer, SRI 8610C SRI Instruments Europe GmbH, was used to measure the oxygen content in gas samples. The HayeSepD column (2 mm ID × 2 m) was installed in the column oven. The column was connected to a thermal conductivity detector (TCD, 157°C) at higher concentrations and a helium ionization detector (HID, 100 V, 204°C) at lower concentrations. A helium gas flow of 48 mL/min was applied, and the column was operated isothermally at 60°C.

## 3 RESULTS

In the results sections “Parallel monitoring of growth and CO_2_” and “Biomass and metabolic state effect on CO_2_ production,” we reveal the correlation between CO_2_ production and classical growth curves, or CO_2_ production and the metabolic activity of the present biomass (one‐phase cultures without oil) of three well known lab strains and single members of the microbial mixture (Table 1), which were employed to simulate the onset of microbial contamination in heating oil storage (two‐phase cultures with oil as the only source of nutrients) in “Assessment of microbial susceptibility of heating oils” section (Figure 2). The correlations were revealed for single strains, but were also active for the microbial mixture.

### 3.1 CO_2_ production correlates with the growth of bacteria, yeasts, and molds

With good oxygen supply in the shaking flask and using species‐specific full media, maximum metabolic activity and maximum CO_2_ production occurred. Therefore, Erlenmeyer flasks aerated via additional vessel connections can be used for CO_2_ measurements. The measured CO_2_ value (mg in headspace) can only be attributed to the currently present and metabolically‐active cells as CO_2_ was not retained in the culture vessel; this allowed the collection of culture samples for classical growth measurements in parallel from identical culture vessels. For *P. fluorescens* and *Y. lipolytica*, optical density and cell numbers were monitored for the duration of the culture. However, for the mold *P. chrysogenum*, growth measurement was limited to the determination of CDW.

**FIGURE 3:**
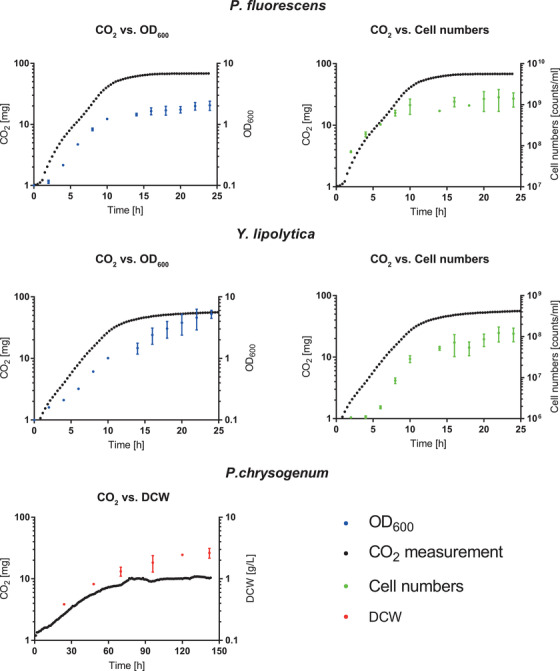
Correlation of CO_2_ development with microbial growth. First row (top), the growth curve of *P. fluorescens*; second row (middle), growth curve of *Y. lipolytica* based on optical density measurement (blue) on the left and cell counting (green) on the right, relative to the measured amount of CO_2_ in 1.22 L headspace (black); and third row (bottom), the growth of *P. chrysogenum* based on the quantification of cell dry weight (red) relative to the measured CO_2_ development. For the growth measurements, mean values of two biological replicates and standard deviations are shown; for the CO_2_ measurement, only mean values of two biological replicates (only one replicate for *P. chrysogenum*) are shown. Both types of measurements are shown on a logarithmic (log_10_) scale

The measured CO_2_ curves resembled the classic microbial growth curves under certain circumstances (Figure 3). The rise and flattening of the CO_2_ curve of the bacterium *P. fluorescens* and the yeast strain *Y. lipolytica* closely followed the time pattern of exponential and stationary phases of developing biomass based on optical density and cell numbers. In contrast, the CO_2_ curve of the mold *P. chrysogenum* reached a plateau after 75 h and the biomass after 142 h. The limitation of the correlation between growth and the CO_2_ signal for *P. chrysogenum* can be attributed to the fact that the CO_2_ signal is not directly dependent on biomass development, but on the metabolic activity of the present biomass. Therefore, the effects of the biomass and the metabolic setting, including non‐growth‐associated metabolic activities, on the CO_2_ signal were considered separately in the following section.

### 3.2 Effect of biomass and metabolic state on CO_2_ formation

**FIGURE 4:**
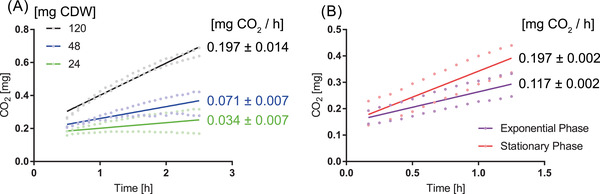
Biomass‐dependent (A) and Growth phase‐dependent (B) CO_2_ enrichment. In A, the first three hours of CO_2_ accumulation produced by 24 (green), 48 (blue), or 120 (black) mg cell dry weight of *P. fluorescens*. In B, the first one and a half hours of CO_2_ accumulation produced by equal amounts of *P. fluorescens* from the exponential (purple) and stationary phase (red). The cell material is always resuspended in 20 mL of a glucose solution and transferred into 100 mL shot flasks for CO_2_ measurement, resulting in 115 mL headspace volume. The dots represent two technical replicates. The solid lines represent linear regressions and are labeled with the calculated slopes (enrichment rates), including standard deviation

The sampling of different amounts of *P. fluorescens* cells, their resuspension in identical volumes of nitrogen‐ and phosphorus‐free glucose solution, and their transfer into airtight shot bottles with the same headspace volume led to proportionally different CO_2_ enrichment rates. A rate of 0.034 mg CO_2_/h was observed for 24 mg CDW, 0.071 mg CO_2_/h for 48 mg CDW, and 0.197 mg CO_2_/h for 120 mg CDW (Figure 4A). As samples were collected from a single culture and a specific growth phase, the metabolic activity was constant at the time of sampling. Differences in the CO_2_ signal were solely due to the different amounts of harvested cells or different cell densities in the same suspension volume. Higher cell quantities were associated with a proportionally higher CO_2_ accumulation rate.

Sampling from the exponential and stationary phases represents two defined metabolic states of a *P. fluorescens* culture. Their resuspension in nitrogen‐ and phosphorus‐free glucose solution and their transfer into airtight shot bottles with the same headspace volume led to a higher CO_2_ enrichment rate by cells in the stationary phase (Figure 4B). The samples of the stationary phase (0.197 mg CO_2_/h) showed a 68% higher CO_2_ accumulation rate than the samples in the exponential phase (0.117 mg CO_2_/h). Disparities in the CO_2_ signal were due to the distinct metabolic activities of the different growth phases. A higher overall metabolic activity in the stationary phase of *P. fluorescens* would be an unexpected result and is thus further discussed.

### 3.3 Application of CO_2_ measurement for the assessment of the microbial susceptibility of heating oils

**FIGURE 5:**
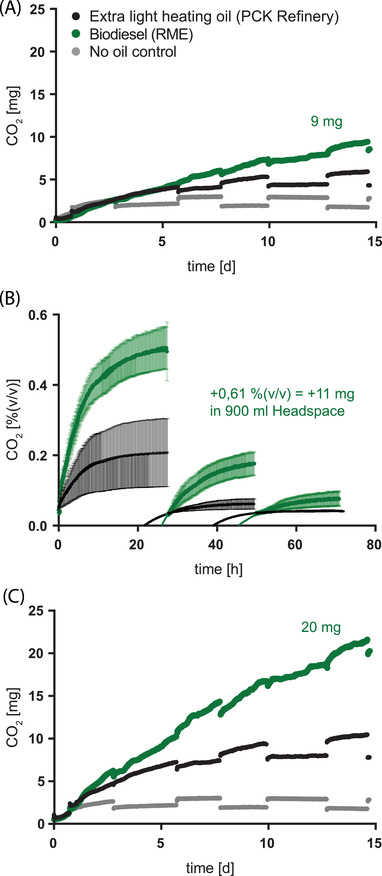
Actual CO_2_ measurement in headspace of storage cultures containing fossil heating oil, biodiesel, or no oil phase (A). Individual values from alternating measurements of the two biological replicates. Degassing of fossil heating oil and biodiesel after completion of the storage cultures (B). Three cycles of degassing and draining of the headspace of CO_2_ in‐between. Mean values and standard deviation of two biological replicates per oil phase are overlaid with a non‐linear (one‐phase association) regression curve. The summed amount of CO_2_ from biodiesel and the sum of the extrapolated values of the regression curves after 1000 hours. Total CO_2_ production as the sum of headspace measurement and CO_2_ stored in the oil phase in storage cultures containing fossil heating oil, biodiesel, or no oil phase (C). Individual values from alternating measurements of two biological replicates

The onset of microbial contamination in a heating oil storage tank with an inoculated free water phase, a common source of microbial contamination [10], was simulated under laboratory conditions and monitored using CO_2_ measurements. A defined inoculum was used according to Leuchtle et al. [14]. For fossil extra light heating oil (HEL), Biodiesel (rapeseed oil methyl ester, RME) and “no oil” control, the accumulation of CO_2_ was monitored over two weeks as a measure of microbial activity. Based on numerous literature sources, biodiesel or blends of biodiesel in fossil heating oil are expected to have a significantly higher microbial activity due to the presence of simpler carbon sources and contamination with additional phosphorus and nitrogen sources [11, 16, 17, 18]. Nevertheless, the amount of microbial CO_2_ measured in the headspace of the culture bottle containing biodiesel could only be insufficiently separated from the amount of CO_2_ measured above the fossil heating oil. The same poor separation was observed between the CO_2_ production above the fossil heating oil and the culture bottle without an oil phase as a source of nutrients (Figure 5A). Degassing of the oil phases after completion of the storage cultures showed that a large part of the microbially produced CO_2_ was stored in the oil phase and therefore could not be measured in the headspace (Figure 5B). In the biodiesel phase, up to 67% of the produced CO_2_ was stored, whereas less than 50% of the produced CO_2_ was stored in the fossil heating oil phases. Therefore, summing up the CO_2_ measured in the headspace and the CO_2_ stored in the oil resulted in a significant improvement in the relative separation of microbial activity among biodiesel and fossil heating oil, especially in the separation of the absolute amounts of CO_2_ produced (Figure 5C). Oxygen consumption was evaluated in a parallel experiment. The initial and final oxygen contents of the headspace were investigated by gas chromatography in a 2‐week storage culture containing fossil heating oil. A decrease from 21% (v/v) to 13% (v/v) oxygen was observed.

## 4 DISCUSSION

### 4.1 CO_2_ production correlates with the growth of bacteria, yeasts, and molds

As expected, the CO_2_ measurements mirrored the course of classical growth curves for the tested bacterium *P. fluorescens* and yeast *Y. lipolytica* (Figure 3, top and middle). For the mold *P. chrysogenum*, at first glance, the course of the CO_2_ curve seems to inadequately reflect growth or fail to reflect growth over the complete cultivation period (Figure 3, bottom). Such result can be explained by an increasing mortality rate, whereby the dead cell material continues to contribute to CDW but not to CO_2_ production, as it is no longer metabolically active [15]. The CO_2_ signal accounts for the increased occurrence of dead, metabolically inactive cell material, resulting in a proper growth curve. The observed deviations in the CO_2_ signal from classical growth, as documented for *P. chrysogenum*, show that the CO_2_ signal is the result of metabolic activity, for which growth can contribute significantly.

### 4.2 Effect of biomass and metabolic state on CO_2_ formation

Crucial for the course of the CO_2_ curve is the metabolic activity of the existing biomass, which has already been indicated in the previous section for the correlation between growth and the CO_2_ signal of *P. chrysogenum*. Irrespective of the decisive influence of the metabolic activity, a linear relationship between the harvested cell quantities (present biomass) of one *P. fluorescens* culture and the resulting CO_2_ accumulation rate was shown for a defined time point of the stationary phase (Figure 4A).

Using constant biomass normalized by optical density, the CO_2_ production of *P. fluorescens* cells during one time point of the exponential and stationary phases, two different metabolic states, was compared. A 68% higher CO_2_ enrichment rate (Figure 4B) was observed for cells in the stationary phase. Therefore, CO_2_ formation is not mainly based on complete metabolic activity, but on the catabolic activity of the cell, such as the activity of the pentose phosphate pathway, amino acid degradation, and citric acid cycle. The higher catabolic activity during the stationary phase is caused by the decrease in competitive biosynthetic reactions, such as lipid biosynthesis or acetate formation. The higher CO_2_ enrichment rate in the stationary phase is also due to the artificial switch from the amino acid‐rich LB medium into a glucose solution, where the catabolic activity of exponential phase cells is suppressed owing to the focus on amino acid degradation. In contrast, cells in the stationary phase may have acquired flexibility regarding the carbon source.

By using nitrogen‐ and phosphorus‐free resuspension solutions, further cell division or an adaptation of metabolic activity could be suppressed during the first hours of measurement, and a linear CO_2_ enrichment could be achieved.

### 4.3 Application of CO_2_ measurement for the assessment of the microbial susceptibility of heating oils

To simulate and track the onset of microbial contamination in an oil storage tank, CO_2_ accumulation was measured over 2 weeks based on inoculated water phases overlaid with a surplus of heating oil or biodiesel (RME) as a nutrient source (Figure 5). Additional storage cultures included no oil phase to quantify oil‐phase‐independent CO_2_ production by the microbes. Over two weeks, the microbial activity measured by CO_2_ accumulation in the headspace reached 2 mg for no oil phase, 5 mg for fossil heating oil, and 9 mg for biodiesel (Figure 5A). Microbial CO_2_ production in the absence of an oil phase is possible because of the use of lysed cells as a source of nutrients. Although a higher microbial activity could be reported for biodiesel than heating oil or for the presence of a fossil heating oil phase relative to no oil phase, greater separation and differences in microbial activity, as indicated by the literature, seem to be technically limited [11, 16, 17, 18]. By degassing the oil phases after the completion of the storage cultures, the oil phases were demonstrated to store large amounts of CO_2_. In addition, biodiesel stores relatively more CO_2_ than fossil heating oil owing to its greater share of polar compounds (Figure 5B). A linear balance between the CO_2_ stored in the oil phase and the CO_2_ released into the headspace was assumed. By comparing the final headspace CO_2_ value and the amount of CO_2_ stored in the oil phase, the headspace CO_2_ measurement (Figure 5A) could be converted into total CO_2_ production (Figure 5C). Over two weeks, the microbial activity measured by total CO_2_ production reached 2 mg for no oil phase, 9 mg for fossil heating oil, and 20 mg for biodiesel (Figure 5A). The test approach, and especially the duration of the test approach, is further limited by the need to prevent gas exchange and oxygen. In additional storage cultures, including fossil heating oil, the decrease in available oxygen from 21% (v/v) to 13% (v/v) over two weeks could be documented by gas chromatography of headspace samples. The low oxygen availability is a decisive difference to that of the real heating oil tank situation and limits the duration of the oil storage tank simulation.

The measuring principle and laboratory format have limitations. Nevertheless, the assessment of the contamination potential of (heating) oil is almost impossible using classic microbial techniques. However, off‐gas analysis provides a simple solution for this demanding sample system.

Finally, the monitoring of CO_2_ accumulation, as a measure of microbial activity, was demonstrated to enable the quantitative assessment of microbial contamination in oil tank conditions without the need for elaborate invasive sampling, and importantly, long before problematic signs, such as biofilm formation, occur. This simple setup can also be easily transferred to other difficult‐to‐monitor conditions, such as plastic degradation by microbes.

## CONFLICT OF INTEREST

The authors declare no conflict of interest.

## Data Availability

The data that support the findings of this study are available from the corresponding author upon reasonable request.
